# Modelling the Effects of Seasonality and Socioeconomic Impact on the Transmission of Rift Valley Fever Virus

**DOI:** 10.1371/journal.pntd.0003388

**Published:** 2015-01-08

**Authors:** Yanyu Xiao, John C. Beier, Robert Stephen Cantrell, Chris Cosner, Donald L. DeAngelis, Shigui Ruan

**Affiliations:** 1 Department of Mathematics, University of Miami, Coral Gables, Florida, United States of America; 2 Department of Public Health Science, Miller School of Medicine, University of Miami, Miami, Florida, United States of America; 3 U.S. Geological Survey, Department of Biology, University of Miami, Coral Gables, Florida, United States of America; Oswaldo Cruz Foundation, Brazil

## Abstract

Rift Valley fever (RVF) is an important mosquito-borne viral zoonosis in Africa and the Middle East that causes human deaths and significant economic losses due to huge incidences of death and abortion among infected livestock. Outbreaks of RVF are sporadic and associated with both seasonal and socioeconomic effects. Here we propose an almost periodic three-patch model to investigate the transmission dynamics of RVF virus (RVFV) among ruminants with spatial movements. Our findings indicate that, in Northeastern Africa, human activities, including those associated with the Eid al Adha feast, along with a combination of climatic factors such as rainfall level and hydrological variations, contribute to the transmission and dispersal of the disease pathogen. Moreover, sporadic outbreaks may occur when the two events occur together: 1) abundant livestock are recruited into areas at risk from RVF due to the demand for the religious festival and 2) abundant numbers of mosquitoes emerge. These two factors have been shown to have impacts on the severity of RVF outbreaks. Our numerical results present the transmission dynamics of the disease pathogen over both short and long periods of time, particularly during the festival time. Further, we investigate the impact on patterns of disease outbreaks in each patch brought by festival- and seasonal-driven factors, such as the number of livestock imported daily, the animal transportation speed from patch to patch, and the death rate induced by ceremonial sacrifices. In addition, our simulations show that when the time for festival preparation starts earlier than usual, the risk of massive disease outbreaks rises, particularly in patch 3 (the place where the religious ceremony will be held).

## Author Summary

Rift Valley fever is a common vector-borne zoonotic disease that causes huge economic losses in Africa and the Middle East. The transmission and dispersal of the disease pathogen are affected by many factors, such as climatic, hydrologic and geographic influences, along with impacts from human activities and different forms of virus transmission via different vectors. In this work, we focus on identifying the potential risks that lead to disease outbreaks in Egypt along the Nile, from the South to the Nile Delta, by mathematically and numerically analyzing a patch model with temporal periodicity. We discover that human activities during the Eid al Adha feast, as well as climatic and hydrological variations, contribute to the transmission and dispersal of the disease. Interestingly, periodic co-occurrence of the religious festival and the onset of peaks in mosquito abundance, each with a different periodicity, is predicted to lead to periodic large scale disease outbreaks.

## Introduction

Epidemics are often a result of two or more risk factors that occur simultaneously. Commonly, in the case of vector-borne diseases, this could be the co-occurrence of high densities of arthropod vectors and large numbers of susceptible individuals in a population. This co-occurrence could happen for a number of reasons. For example, some combinations of climatic factors such as rainfall level that may favor the growth of a vector population, and human activities, such as events involving large congregations of hosts in one place, could occur together around the same time, perhaps periodically. This enhances the risk that a single case of a disease will rapidly spread to an epidemic. Often, it is not possible to predict such co-occurrences. However, in some cases, such temporal superposition of risk factors can be predicted well in advance. We discuss such a case here using a simple model for Rift Valley fever virus (RVFV), a vector-borne pathogen endemic in Africa and the Middle East. In particular, the case investigated here involves the periodic coincidences of a natural phenomenon, annual flood stages of a river, which promotes high densities of disease vector mosquitoes, and a religious festival, the Eid al Adha feast, at which time large numbers of livestock are driven towards the site of the feast. It creates the particular periodicity of times of high potential for disease outbreaks, RVFV in this case, because the river flood stage follows the solar (365.25 days) calendar, whereas the religious feast follows the lunar calendar (354.37 days). This means that these two events will coincide perfectly only every 33.57 years, although partial overlap occurs in other year surrounding those of perfect coincidence, depending on the durations of both the high flood stage of the river and the festival. Drake et al. [1] conducted a statistical model to investigate the influence of these two events on the disease outbreaks. This is analogous to what is known in acoustics as a “beat frequency”; e.g., when a piano is out of tune and two strings belonging to the same note are not vibrating at exactly the same rate, a quavering will occur with a frequency of the difference of the frequencies of the two strings.

RVFV is a type of viral zoonosis that is primarily transmitted among animals, including cattle, sheep, goats, and camels, via bites from female mosquitoes. Humans are also hosts for this virus and severe human infections are caused by direct or indirect contact with the blood or organs of infected animals. However, humans are dead-end of the transmission of RVFV, as they will not cause new infections via bites among mosquitoes. This disease has drawn substantial attention, as it can cause significant economic losses due to huge incidences of death and abortion among infected livestock. In the 1930s, RVFV was recognized in the literature [2]–[4] as a disease primarily of the southern part of Africa. During the following two decades, it expanded to countries such as Zimbabwe, Nigeria, and Chad [5]. In the 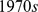, the first human infection was reported in Egypt [6]. After that, the disease invaded Saudi Arabia and Yemen [7] in the early 2000s. This epizootic has thus spread from southern Africa to North Africa, and beyond to the Middle East and Madagascar [8], [9]. RVFV has shown its ability to invade ecologically diverse regions and has eventually spread throughout the entire continent of Africa [10]. Geographically, it seems that the disease has followed a path from southwest to northeast in Africa. Interestingly, the invasion path in Egypt follows the same route that some Egyptians use to travel to the Nile Delta, where the important Islamic festival Greater Bairam is held. This path that we are modeling is also partially coincident with the path by which Egyptians travel to Mecca, the capital city of Saudi Arabia.

Between 1950 and 1976, at least sixteen major outbreaks of RVFV occurred among livestock at various locations in sub-Saharan Africa (see Table 1). In Egypt, there have been five major outbreaks among humans in the past four decades [1]. The first two major RVFV outbreaks occurred during the period of July to December in 1977 and 1978. Fifteen years later, there was another RVFV outbreak among humans from May to July in 1993 [11]. Although there is little data available for the infections among livestock, it is reasonable to believe that the disease was also prevalent among domestic ruminants. It is noted in [12] that “During an epidemic, the disease usually occurs first in animals, then in humans”. Between April and August in 1997, a fourth RVFV outbreak caused an extensive epizootic of RVFV in Egypt. The high morbidity and mortality rates in domestic ruminants led to an official report concerning an outbreak of RVFV among livestock in Egypt [6], [13], [14]. The most recent outbreak of the disease in Egypt occurred between June and October, 2003, causing around 375 human cases. The five major outbreaks coincided with either the peak season of mosquitoes in Egypt (July-September, 1977 and 1978, 2003) or the timing of Greater Bairam, Eid al Adha feast (October-December, 1977 and 1978, 1993, 1997). Many researchers have noticed this interesting phenomenon and have attempted to use mathematical and statistical models to identify the underlying relevance [1], [10], [15]–[23]. A mathematical model considering two species of mosquitoes as vectors was analyzed in [10], and [16] presented a patch model to investigate the effect of livestock movement on the spread of RVFV. In [18], [19], a network based model was used to evaluate the spatial dispersal of RVFV, while Drake et al. [1] examined a statistical model to identify the potential risks for disease outbreaks, which is the motivation of our work to further explore the how these risks will impact disease dynamics.

**Table 1:** Summary of outbreaks of Rift Valley fever in Egypt, 1977–2011 [1].

Outbreak year	Month	Primary Epidemiological References
1977, 1978	July-December	Meegan [6]
		Darwish 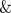 Hoogstraal [51]
1993, 1994	May-July	Arthur et al. [11]
1997	April-August	Abd el-Rahim [33]
2003	June-October	Okda et al. [12]
		Hanafi et al. [52]

The reasons for the disease outbreaks among livestock have been explored for quite a long time. One of hypotheses is that in Egypt, Saudi Arabia, and Yemen, outbreaks may occur when the disease is introduced by the importation and transportation of infected animals [24]–[26]. Egypt had been Sudan's main trading customer [27]. In 1989, Saudi Arabia became Sudan's main export market, buying an estimated 16.8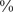 of Khartoum's exports, particularly sorghum and livestock [27]. In the past three years, statistical data from the Egypt Livestock and Products Annual Report 2013 [28] show that almost 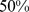 of human consumption of meat is from imported meat. When the largest Islamic festival, Eid al Adha, approaches, the ceremonial sacrifices drastically increase the demand of livestock. For example, most live cattle imported mainly from Brazil, Sudan, Ethiopia, Croatia, and Australia are earmarked for immediate slaughter [28]. It is estimated that 1 to 2 million animals were sacrificed a day during the festival [29]. One of the domestic newspapers, Egypt Independent, published a news report on Tuesday, September 17th, 2013, that lobbied the government to increase meat imports ahead the Eid al Adha feast. Many researchers have been questioning the hidden side-effects of the flourishing border trade [1], [17], [24]. Davies [29] believed that the spread of RVFV among ruminants was accelerated by imports of livestock from Sudan to Egypt, and further increased by the religious festival, Eid al Adha. In order to arrive at the rendezvous, i.e. the Nile Delta, where millions of Muslims celebrate the Feast by the ritual sacrifice of a ram by ‘halal’ after long-distance travel, people travel by foot or train. One of the ground routes starts from Sudan, then follows the Nile in Egypt from south to north, and further traverses from northwest to southeast in Saudi Arabia along the coastline of Red Sea [29]. The ceremonial sacrifices lead millions of livestock to follow the paths of the Muslim pilgrimage, on foot, by train or by ship depending on the travel distance [24], [30], [31]. It was reported that “More than one million sheep, 750,000 from Somalia and 350,000 from Sudan, will be imported by the Al-Jabri Company, the main provider of the Saudi Project for Utilization of Sacrificial Animals” in the Saudi Gazette [32].

On the other hand, ceremonial sacrifices will lead to a much higher death rate for livestock (mainly cattle and sheep) in the Nile Delta compared with the other locations during the festival. This may also affect pathogen transmission, as the high rate of slaughter will increase the demand for the importation. According to some literature noted in the preceding paragraph [24]–[26], it is believed that importation of livestock in Egypt from Sudan and other countries, transportation of livestock following the pilgrimage, and the death rate of livestock in the Nile Delta will be impacted by the annual festival according to the lunar calendar. In this paper, we use the movement speed of livestock and length of journey for animals within a patch to measure the level of transportation; i.e., the increase of the volume of livestock transportation will be exhibited by the increase of animal movement, with fixed length of journey within each of the patches modeled. During the festival season, the values of these parameters will keep rising until the day of Eid al Adha, and then decrease to their baseline levels at other times. To mathematically evaluate the hidden danger of the Feast and the trade driven impacts of livestock, we use the fact that the number of livestock imported in Egypt, the movement speed of livestock, and the death rate in Mecca follow a periodic pattern, where the period is the length of a lunar year (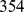 days).

Statistical research has shown a relation between the carrying capacity of mosquitoes and local climatic factors, such as the weekly temperature, amount of rainfall [1], [33], total temperature degree-days [34], and the temperature of water. Many species of mosquitoes found in Africa have been reported to be the vectors of RVFV, such as *Aedes caspius*, *Culex(Cx) pipiens*, *Cx. antennatus*, *Cx. perexiguus* and *Cx. poicilipes*. An abundance of mosquitoes may be found along the Nile, in its Delta and near the Suez Canal. Egypt is an arid country with a hot desert climate, where the most commonly reported mosquito species are *Culex* species [35]. Unlike the *Aedes* in places like Kenya [36], *Culex* species are not known to be involved in the endemic maintenance of RVFV by vertical transmission. In this work, we focused on the pathogen transmission in Egypt, therefore, we only consider the *Culex* species as vectors in our mathematical model for transmission of RVFV in Egypt and ignore the possibility of the laying of virus-laden eggs by the species *Aedes caspius*. Of the reported species, seasonality was observed in all species, particularly the two most common species: *Cx. pipiens* and *Cx. perexiguus*. Both species are observed all year round, but have significantly higher densities during April/May to September/October, coinciding with high water levels and temperatures [37]. Therefore, it is biologically reasonable to believe the abundance of mosquitoes follows the seasonal patterns of the level of Nile, peaking in mid-September [38] and reaching lowest level during the winter season [1]. Motivated by the field curves for rainfall level in [1], we adopt a trigonometric function to model the seasonal variation of rainfall level. As a consequence, since the mosquito abundance follows the same pattern of rainfall, we posit that the carrying capacity of the vector is also a trigonometric function, where the period is the length of one solar year, 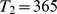 days. In this paper, for the sake of simplicity, we use the function 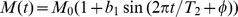 to model the seasonal variation of the mosquito carrying capacity, where 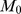, 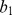 and 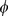 are the baseline of carrying capacity, amplitude and phase, respectively. We start our simulations from January 1st, 1977; therefore, we have the phase chosen as 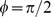 so as to start with a low mosquito abundance. Due to the lack of field data specific to the situation we are modeling for mosquitoes, we could not get true values for baseline of mosquito carrying capacity 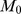 and amplitude 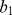.

The purpose of this paper is to evaluate the seasonal and festival-driven impacts on RVF outbreaks and spatial dispersal. In the section Materials and Methods, we derive an ordinary differential equation system with periodic drivers to mathematically model the transmission and dispersal of RVFV, and followed by numerical simulations in section Results. We provide some fundamental mathematical analysis on the model in S1 Text.

## Materials and Methods

In the last a few years, various mathematical models have been developed to study the transmission dynamics of RVFV [10], [17], [21], [22]. Gaff et al. [10] investigated a model to capture the two mechanisms of RVFV pathogen transmission by both *Aedes* and *Culex* mosquitoes; vertical transmission via infected eggs among species *Aedes* and indirect transmission via mosquito biting by both species. In Gao et al. [17], a three-patch model was employed to model the directional livestock movement. In both of these papers, the basic reproduction number for stability analysis of equilibria with constant coefficients was derived. In this work, we follow the idea in [17], and construct a three-patch model with some periodic coefficients replacing the previously constant parameters to investigate the effects of seasonality and socioeconomics on the transmission of RVFV in African and the Middle East.

### Single patch model

We divide the total population of livestock into three classes: susceptible (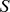), exposed (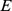), infected (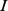), and recovered individuals (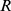). The female mosquitoes have two subgroups: uninfected (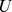), exposed (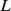) and infected (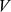). Since the abundance of mosquitoes is very sensitive to local temperature and the level of rainfall, which undergo annual cycles, we have a periodic function 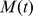 representing the carrying capacity of mosquitoes. The transmission of the disease follows the typical mechanism for vector-borne diseases [39], where we use a SEIRS structure for livestock and a SEI format for vectors. Due to the sudden concentration of the host population during the short period time (celebration period) by the importation or transportation of animals driven by the religious festival, the biting rate will quickly increase assuming the hungry mosquitoes are abundant in each patch. Therefore, we assume that the effective biting rate is linearly dependent on the livestock population (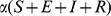). Further, we have the new infection term 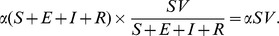 Since the pseudo-mass action mechanism works appropriately when disease transmission occurs in discrete, compact colonies (i.e. farms in our case) [40], [41]. Here we employ this transmission mechanism to describe the infection force for Rift Valley fever. Therefore, a single patch model for RVFV transmission is expressed as follows:
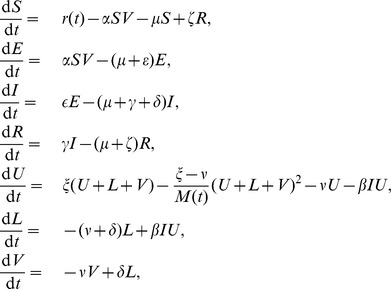



Here, 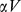 (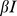) is the force of infection when the disease pathogen transmitted from mosquitoes to livestock (livestock to mosquitoes); 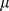 (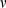) is the natural death rate for livestock (average death rate for different livestock, i.e. cattle and sheep) and female mosquitoes, respectively; 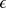 (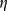) is the rate of becoming infectious for livestock (mosquitoes), respectively; 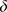 is the disease-induced death rate for livestock; 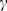 is the recovery rate from infection; and 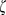 is the rate at which immunity is lost after recovery (livestock). Here, the logistic growth is adopted to describe the birth and death processes for mosquito population in the fourth and fifth equations of system (1), that is the total number of mosquitoes (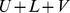) follows the logistic equation, where 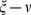 is the growth rate of the total population and 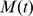 is the time-dependent carrying capacity of mosquitoes. One could obtain the logistic equation for the total mosquito population by simply adding the equation of 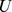 and 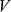 together. 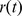 denotes the number of livestock imported daily, which is time-dependent according to the assumptions in the previous section (i.e. periodically associated to the lunar calendar).

This model is similar to the Ross-Macdonald model. By introducing a next generation operator [42], we can obtain its basic reproduction number 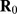, which considers the variation of parameter values over a period but is independent of the time variable. Unfortunately, we could not obtain an explicit form of 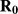. Nevertheless from [42] and [43], we have the following analytic result: when 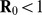, the disease will die out eventually, whereas when 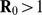, the system admits a positive solution (endemic state). In order to examine the disease dynamics at some particular time within a season, we can also compute the *instantaneous basic reproduction number* via the method of next generation matrix [44], [45], wherein
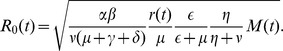



From the above expression, one can see that the instantaneous 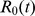 is computed based on the values of the function 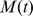 and periodic parameter 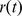 at time t. Although the value of 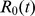 is time-dependent and cannot be used to predict the disease outbreak over one period, it does precisely capture the pattern of disease dynamics at that particular time. As we are focusing on the impact of livestock movements on disease dynamics, we extend our one-patch model to a multiple-patch model. Since we could not even get an explicit form for 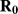 for the simple model (1), we cannot expect to investigate the relations between key parameters, i.e. importation level, and 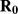. Therefore, in the rest of the paper, we will focus more on the instantaneous basic reproduction number and use it as a threshold to predict the disease dynamics during the festival season.

### Multiple patch model

**Figure 1:**
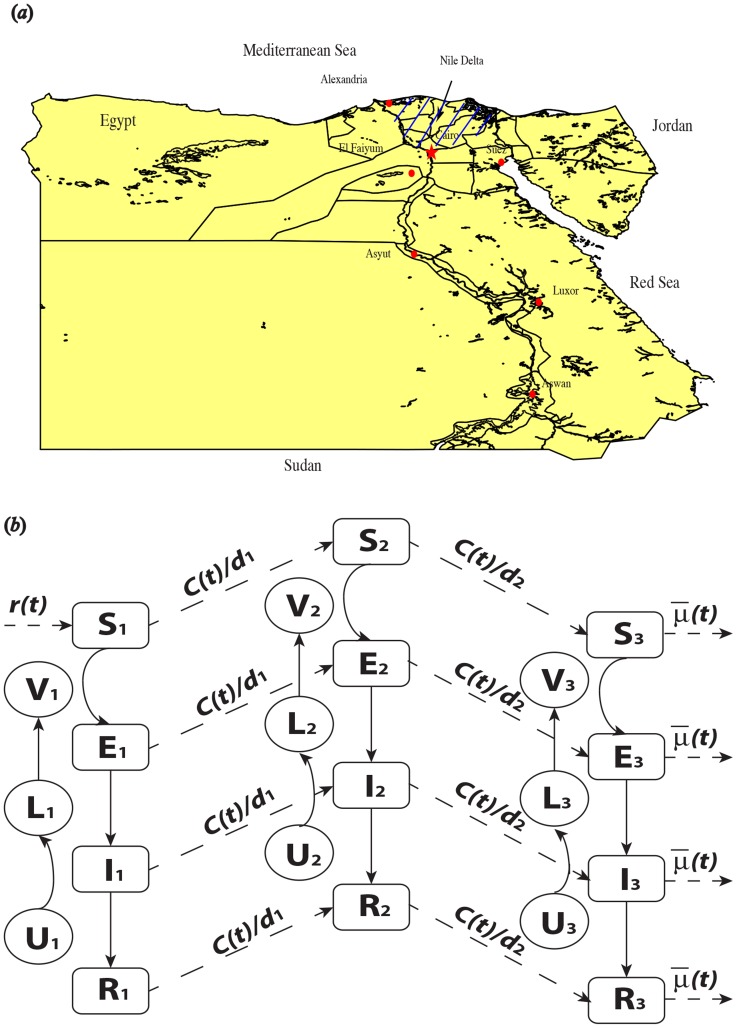
Map of Rift valley fever in Egypt and flow chart. (a) Map of Egypt; (b)The flow chart of RVFV transmission and spatial dispersal. The sub-script 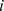 represents the related compartment in patch 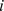, and the other parameters are listed in Table 2. Only the first patch has import of livestock, and then livestock, regardless of infection status, move from patch 1 to patch 3, via patch 2 with human demand. Within each of the patches, the disease pathogens are transmitted between livestock and mosquitoes causing infections. Directions in dash represent the seasonally or socioeconomically driven flows.

Although RVF causes human infections as well, we do not consider humans directly in our single patch model. Infected humans will not transmit the RVFV to other humans or back to vectors, as they are the dead end of the pathogen transmission. Adding human compartments increases the total number of infected individuals, but it does not change the transmission dynamics of RVFV between mosquitoes and livestock. On the other hand, human movement and behavior do affect the disease dynamics by regulation of livestock importation level, movement speed, and death rates (including the rate of slaughter) in each patch. Therefore, we incorporate human activities via the periodic parameters for import level, movement speed, and death rate of livestock. The spread of RVFV is also believed to link with regional human dispersal in Egypt. To model the spread of the disease along the Nile or the coastline of Red Sea (from southern Egypt to the Nile Delta and near the Suez Canal) [46], we adopt a directional three-patch model (refer to Fig. 1 (b)), similar to the idea of the three-patch model for RVFV studied in [17], and further extend the model by adding exposed classes for both host and vector population in each patch. Patch 1 represents region from the boarder between Sudan and Egypt to Aswan, patch 2 consists the land of the long region along the Nile (Aswan to Cairo) or the long coastline of Red Sea, and the last patch covers the region of the Nile Delta (shaded area in Fig. 1 (a)). Denote by 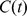, the movement speed of livestock in all groups, regardless of infectiousness, from patch 1 to 2 and 2 to 3. As the Nile Delta is modeled by the last patch, the death rate 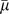 is periodic in patch 3. In particular, there is barely any outflow of livestock from patch 3, as Mecca is a city whose economy has been heavily dependent on the annual pilgrimage [47] and thus export few livestock. Therefore, we ignore the movement of livestock and consider only a periodic death rate in patch 3. In this paper, we use only a three-patch model, but the number of patches can be increased based on geographic divisions. However, since the overall qualitative behavior of the system does not change by adding more patches, without loss of generality, we present a three-patch model in the paper.

**Table 2:** Parameters in the model.

Parameters	Explanations	Units
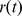	Number of livestock daily imported to patch 1	per day
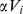	Force of infection when the disease pathogen transmittedfrom mosquito to livestock for patch 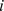	-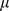	Death rate of livestock in patches 1 and 2	per day
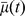	Death (removal) rate of livestock in patch 3	per day
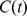	Average moving speed of livestock within patches 1 and 2	km per day
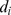	Length of journey for livestock in patch 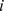	km
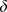	Disease induced mortality rate for livestock	per day
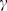	Recovery rate for infected livestock	per day
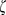	Rate of losing immunity after recovery for livestock	per day
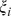	Propagation rate for mosquitoes in patch 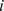	per day
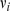	Natural death rate for mosquitoes in patch 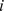	per day
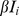	Force of infection when the disease pathogen transmittedfrom livestock to mosquito for patch 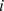	-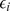	Rate of becoming infectious for livestock in patch 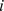	per day
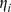	Rate of becoming infectious for mosquitoes in patch 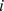	per day
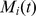	Carrying capacity for mosquitoes in patch 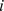	-

Assuming passing the boarder of two patches is instantaneous, therefore births and deaths of livestock are negligible at that moment, the model is
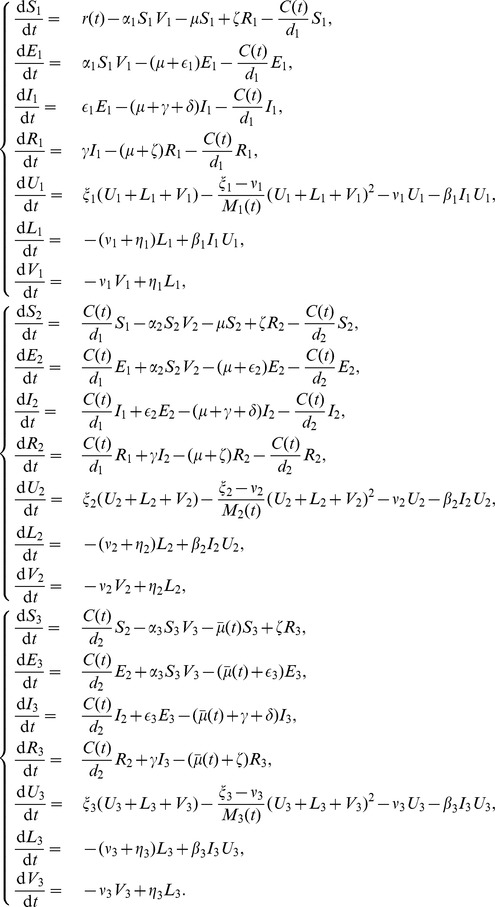



Here, vectors are assumed to be restricted within their own patch due to the limitations of their flying distance.

We assume that functions 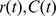 and 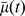 are positive Lipschitz continuous periodic functions, in the sense that for every 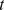 in 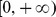 there exists a neighborhood 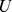 of 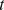 such that 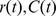 and 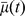 restricted to 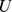 is Lipschitz continuous, i.e. 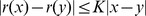 where 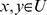 and 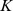 is a constant, mappings from 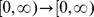, and hence are bounded. Here, due to the periodicity, 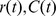 and 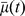 are actually globally Lipschitz continuous. These three parameters vary with the period of a lunar year, where the carrying capacities of mosquitoes in each of the patches 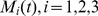, are also periodic but vary with the period of a solar year. The model is developed to examine the influence of the difference between lengths of the two periods on the varying probabilities of transmission of the disease pathogen over periods of many years.

## Results

In this section, we examine the impact of periodic parameters on the dynamics of infectious populations of livestock and female mosquitoes, and investigate how they can affect the instantaneous reproduction number in numerical simulations. The values of most constant parameters are adopted from references [10], [17], [48].

**Figure 2:**
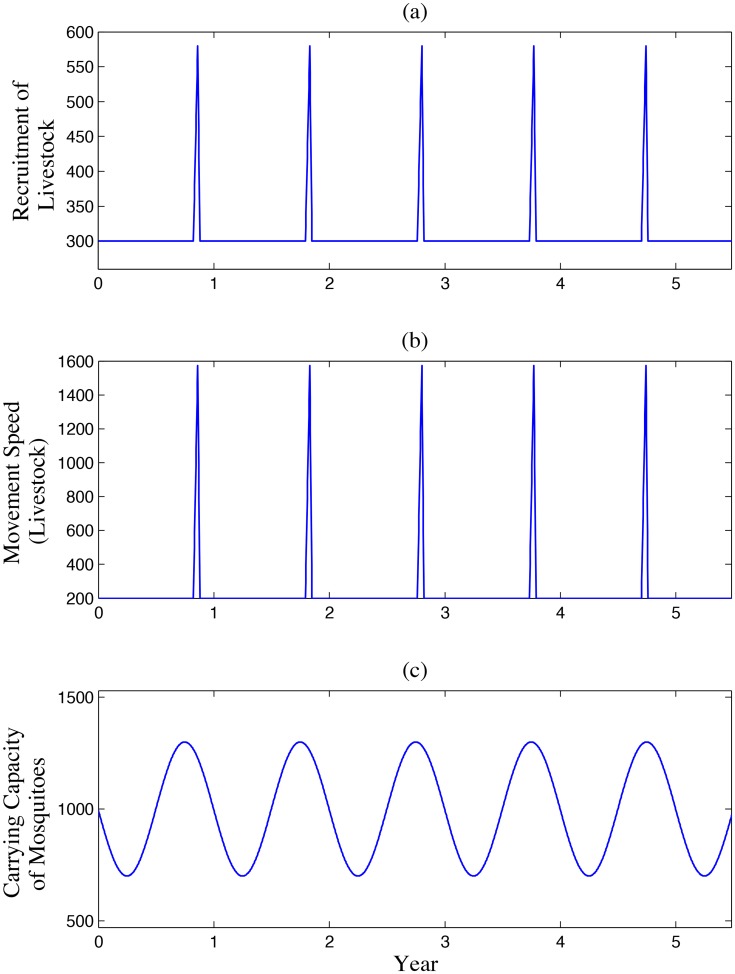
Periodic parameters. (a) The number of livestock daily imported 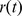, 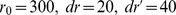 (per day); the peaks represent additional livestock recruited for the feast above the background level 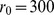, and are related to the lunar calendar. The removal rate of livestock in patch 3 has similar curve. (b) capacity of mosquitoes 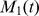, 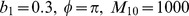. Unit: daily.

In the previous section, we discussed properties of the periodic parameters: the number of livestock daily imported to patch 1, 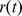, the movement rate of livestock from patch 1 to 2 (2 to 3), 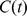, and the removal rate of livestock in patch 3, 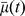. Based on historical records concerning the import of livestock and festival activities in [1], [24], we assume that the number of livestock imported to patch 1 will increase daily by an increment 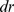, starting at a time 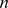 days ahead of the festival (the preparation of the festival started 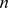
days ago). The daily imported number of livestock reaches its peak on the day of Eid al Adha, and gradually decreases to the baseline level within 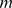 days at a daily rate of 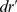. Then, we have 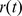 represented by:
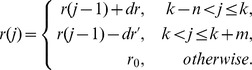
where 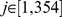 and the *k*th day is the day of Eid al Adha, which varies by the selection of the initial simulation time (i.e. 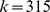, if the simulations start on January 1st, 1977). 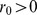 is the baseline for the number of livestock daily imported to patch 1. Due to the lack of field data, we estimate 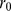 based on references [17], [24]–[27]. Similarly, we can write functions of the other two periodic parameters: 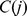 and 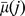 with daily increments 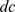 and 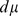 (decrements 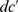 and 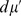). Curves of some selected parameters are presented in Fig. 2 (a)-(c).

**Figure 3:**
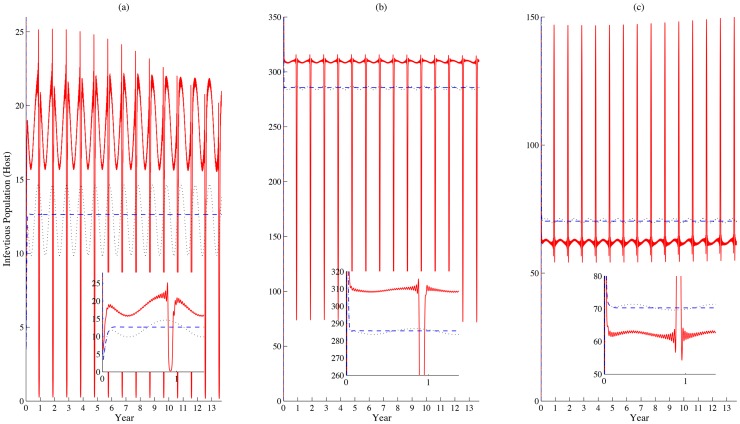
Model comparison. (a), (b) and (c) show the populations of infectious livestock in patches 1, 2 and 3, respectively. The dashed, dotted, and solid lines represent three scenarios with 1) no periodic factors; 2) only the capacity of mosquitoes is periodic; and 3) parameters incorporating both seasonal and festival impacts. Values of parameters: 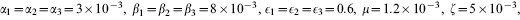 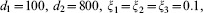 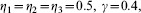 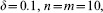 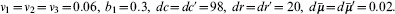 Initial conditions: 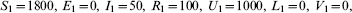 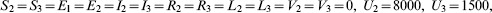 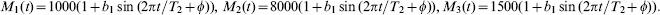 Units: daily.

The seasonal and festival-driven parameters lead to periodic patterns for both infectious host and vector populations. One can easily observe that when all parameters are constant and the carrying capacity of mosquitoes is fixed at its background level, the size of infectious livestock population in each patch converges to a steady state, as in Fig. 3 (a)-(c). When the seasonal pattern of mosquito carrying capacity is included, we observe that a mild periodicity occurs. When the festival-driven impacts are added, we find that the populations of infectious livestock in each patch have a lunar periodicity with much greater amplitudes. During the festival period, with a faster movement speed, more livestock from each group will be transported from patch 1 to 2 (2 to 3); therefore, there are sudden drops of infections in patches 2 and 3. Meanwhile, infected livestock concentrate in patch 3, leading to an increase of infected population size first, but then mass slaughter for the feast (festival-driven death rate of livestock in patch 3) reduces the number of infected individuals quickly, shown in Fig. 3(c). Although we assume no livestock stay in patches 2 and 3 initially (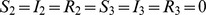), the directional connections between patches bring livestock to these two patches. In particular, the overall population size of livestock in patch 2 is the biggest, as patch 2 is hypothesized to be the longest among the three patches (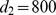).

**Figure 4:**
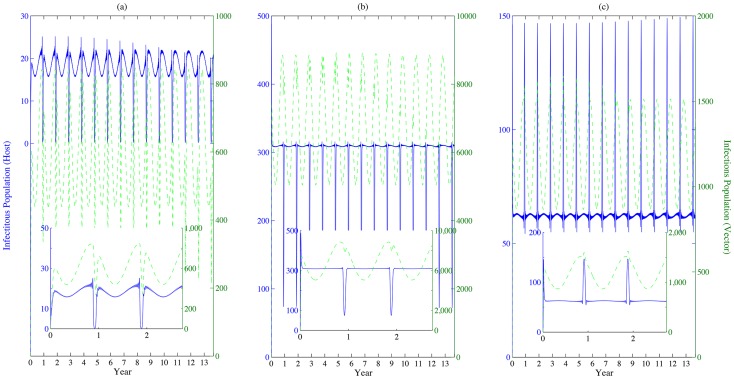
Populations of infected livestock and mosquitoes. (a), (b) and (c) represent the populations of infectious livestock (solid line) and vectors (dashed line) in patches 1, 2 and 3, respectively. Same values of parameters are adopted in Fig. 3. Populations of both infected livestock and mosquitoes alter their patterns during the festival time. Due to the effect of increased movement rates during the festival, the peak of infected livestock population is not necessary to be the same as that of the infected mosquito population, i.e. patch 2.

**Figure 5:**
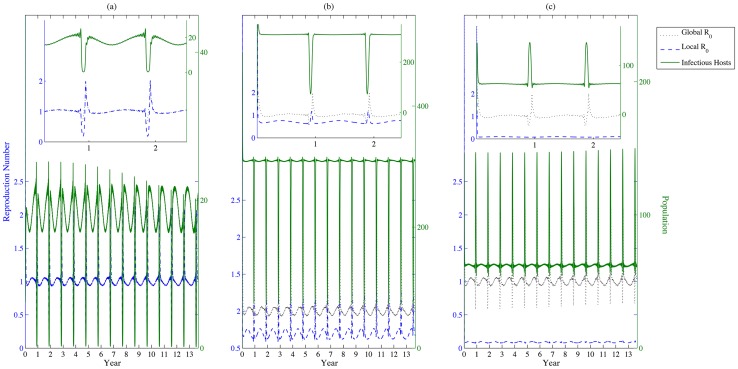
Basic reproduction number. (a), (b) and (c) represent the populations of infectious livestock (solid line), the instantaneously local basic reproduction number (dashed line) and the instantaneously global basic reproduction number (dotted line) in patches 1, 2 and 3, respectively. Values of other parameters are identical with those in Fig. 3. The instantaneously global basic reproduction number is computed by considering the three patches as an entirety, while the instantaneously local basic reproduction number is measured only within the local patch based on the current disease dynamics.

We further compare the infected population sizes of both livestock and mosquitoes. We find that the peaks of the infectious populations for both livestock and mosquitoes are coincident in patch 1 in Fig. 4 (a). However, these two peaks do not appear at the same time in patches 2 and 3; i.e. in Fig. 4(b), the peak of infectious livestock population emerges when the size of the infectious mosquito population approaches its minimum. This phenomenon may be the consequence of the oscillating inflows of infected livestock from the other patches. The outbreaks of RVF in patch 1 lead to the occurrence of disease outbreaks in patches 2 and 3. Even if the local basic reproduction number in patch 3 is less than 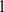, the disease will still persist, as shown in Fig. 5 (c).

**Figure 6:**
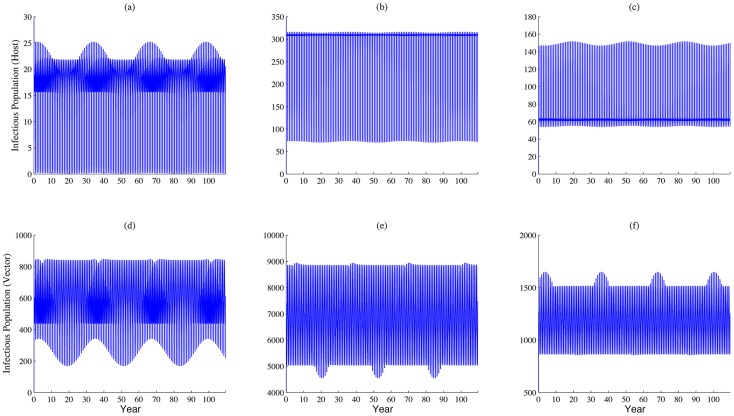
Long term dynamic of disease outbreaks. (a), (b) and (c) represent the populations of infectious livestock, (d), (e) and (f) represent sizes of the infectious mosquito population in patches 1, 2 and 3, respectively. Values of parameters are the same as those used in Fig. 3. We can observe that there is a large scale of disease outbreak around every 30 years, within the festival season.

Long term patterns of infectious population size simulations are shown in Fig. 6 (a)-(f). One can observe that the scale of disease outbreaks peaks every three to four decades. This is due to the coincidence of abundance of mosquitoes related to solar calendar and high number of livestock during religious lunar festival. The large population sizes of both hosts and vectors provide a suitable environment for transmission and spread of the disease pathogen. Since the difference between solar and lunar calendars is roughly 11 days, the coincidence occurs approximately every 33 years (by the solar calendar).

**Figure 7:**
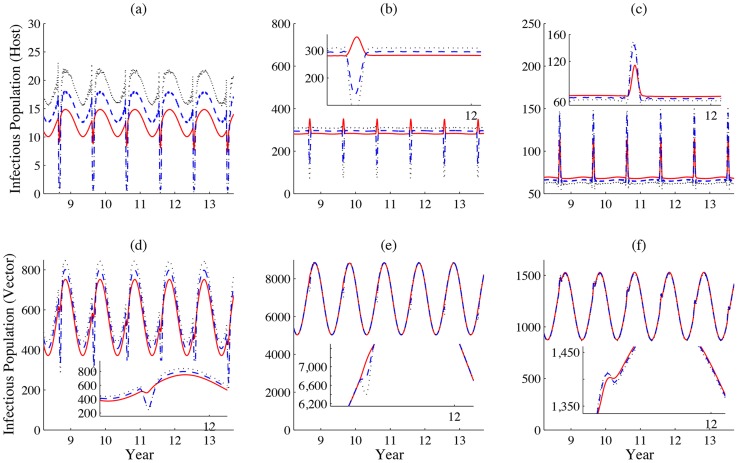
How daily increment in movement speeds impacts patterns of disease outbreaks. (a)-(c) show the populations of infectious livestock and (d)-(f) simulate the populations of infectious vectors in patches 1, 2 and 3, respectively. The daily increment of movement speeds are 10, 54 and 98 km/per day, represented by solid, dashed and dotted lines, respectively. Values of other parameters are identical with those used in Fig. 3. During the festival season, infectious livestock are transported from patch 1 to patch 3, via patch 2, therefore we can observe that the population of infectious livestock has a sudden drop in patches 1 and 2 while an increase in patch 3 in the case that movement speed is relative fast (the increment 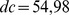).

**Figure 8:**
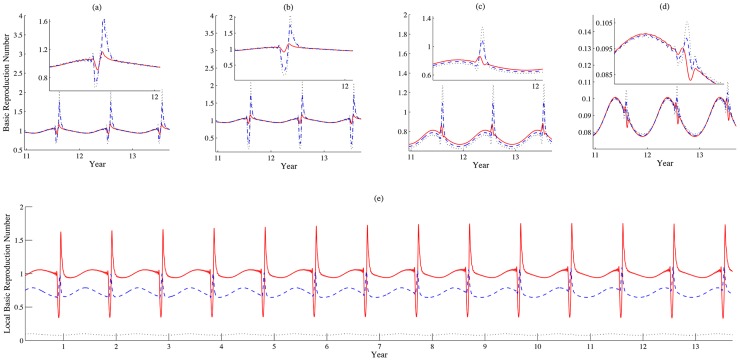
How daily increment in movement speed impacts basic reproduction numbers. (a) Instantaneously global reproduction number; (b)-(d) Instantaneously local reproduction numbers in patches 1, 2 and 3. In (b)-(d), 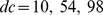 km per day, described by solid, dashed and dotted lines, respectively. (e) Local reproduction number in patches 1, 2 and 3 are simulated by solid, dashed and dotted lines when 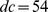 km per day. Values of other parameters are identical with those used in Fig. 3.

Next, we explore how the three periodic parameters, the number of livestock daily imported to patch 1, the speed of movement from patch to patch, and the removal rate in patch 3 (particularly the first two), affect the dynamics of infectious populations. The faster the movement speed of livestock is, the more infected animals will be transported from patch 1, via patch 2, to patch 3. In Fig. 7 (a)-(f), one can observe that when the daily increment in the movement speed (livestock) increases, the size of daily infectious host or vector populations decreases during the festival time in the first two patches, particularly in patch 1; meanwhile, sizes of daily infectious populations increase in patch 3. Except for the festival period, the sizes of infectious populations remain the same in patches 2 and 3 respectively, regardless of the variation in 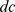. When the increase of the movement rate during the festival time is mild (i.e. 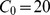
km/day and daily increment of movement speed 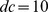
km/day), then infected ruminants will accumulate in patch 1, and the number of daily infected individuals will temporarily increase, shown by the solid curve in Fig. 7(b). With a higher daily movement (transportation) rate during the festival period, more livestock from each group will move from patch 1 to patch 2 and patch 2 to patch 3 in a short time, which leads to the temporal drop of the daily counts of infected individuals in patches 1 and 2. These infected individuals concentrate at their travel destination, patch 3; therefore, the daily count of infections in patch 3 increases quickly during this period. As a further consequence, the total number of infected mosquitoes in each patch follows the same pattern of the infected livestock within the same patch (shown in Fig. 7 (d)-(f)). A relationship can also be observed between the local reproduction number and 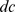. The advances year-by-year in the dates of the within year peaks are also due to the discrepancy between the solar and lunar periods. The oscillation of the local reproduction number has a higher amplitude with a larger 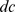 (Fig. 8 (a)-(d)). The local basic reproduction number relies on the size of the susceptible population. The directional movement of livestock will also lead to a shrinking pool of the susceptible population, so the local basic reproduction numbers fluctuate widely. One can observe decreasing curves of local reproduction numbers when the day of Eid al Adha is approaching, and sharply rising curves after the day of the feast. The significant change of the susceptible pool leads to an even higher level of the local reproduction number compared to that brought by a moderate change (slow movement), but this effect diminishes from patch 1 to patch 3 due to the movement of infectious population, shown in Fig. 8 (e).

**Figure 9:**
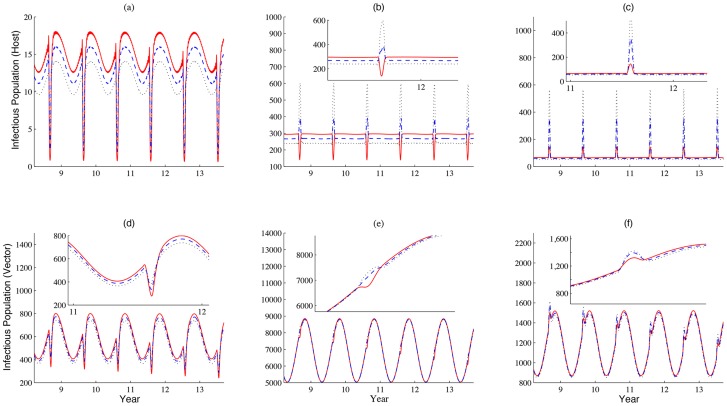
How daily increment in the daily imported number of livestock impacts patterns of disease outbreaks. (a)-(c) and (d)-(f) show the populations of infectious livestock and vectors in patches 1–3, respectively. The increment 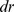 on the daily imported number in patch 1 are 20, 100 and180 per day, represented by solid, dashed and dotted lines, respectively. Values of other parameters are identical with those used in Fig. 3 and 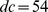 (km per day).

When we fix the movement speed of livestock during the festival period, and vary the number of animals daily imported to patch 1, we find that a bigger number of daily imported livestock during the festival season brings a larger size of infectious livestock population (Fig. 9 (a)-(c)). However, if the number of daily imported livestock during the festival time is increased slowly, then the effect brought on by the increased transportation of livestock dominates, and there will be sudden drops of daily infected population in patches 1 and 2, shown by the solid curves in Fig. 9 (a) and (b).

**Figure 10:**
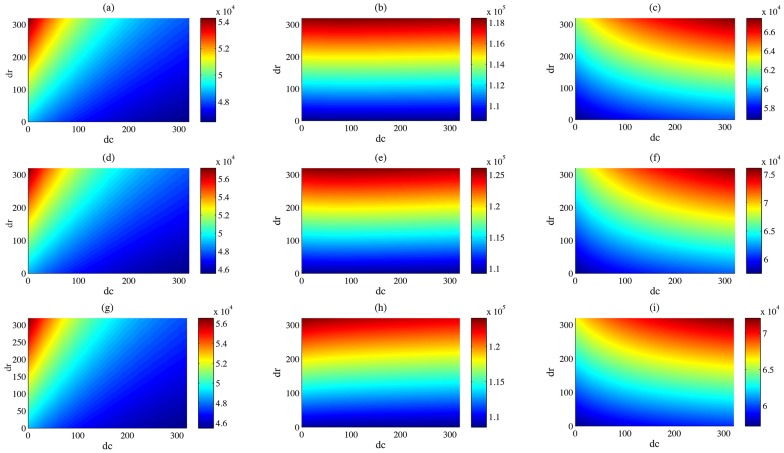
Interaction between the daily increment in movement speeds and the daily imported number on the size of cumulative infected livestock population. (a)-(i), simulations of the cumulative numbers of infected livestock at year 4, 29, and 62 (by row) in patches 1, 2, and 3 (by column). Same values of parameters used in Fig. 3.

Further, we use the yearly cumulative infected population size of livestock to evaluate the annual scale of the disease outbreak. Although the effects brought by variations of the daily imported number and the livestock movement rate during the festival period on yearly cumulative infected population size are much milder compared with that on the daily infected population size, we can still observe that when the increment in the daily imported number of livestock 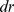 decreases or the movement speed 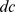 increases, the scale of the disease outbreaks decreases in patch 1 (Fig. 10 (a), (d) and (g)); however, the impact of livestock movement speed on cumulative infected livestock in patch 2 is negligible (Fig. 10 (b), (e) and (h)). Both rises of livestock movement speed and daily imported number of livestock will enhance the scale of RVF outbreaks in patch 3 (Fig. 10 (c), (f) and (i)). The coincidence of two events, 1) a larger size of livestock population flow into patches due to festival demands, 2) appearance of abundant female mosquitoes when the level of the Nile raises, leads to a larger size of the cumulative infected population (Fig. 10 (d)-(f) and (g)-(i)).

**Figure 11:**
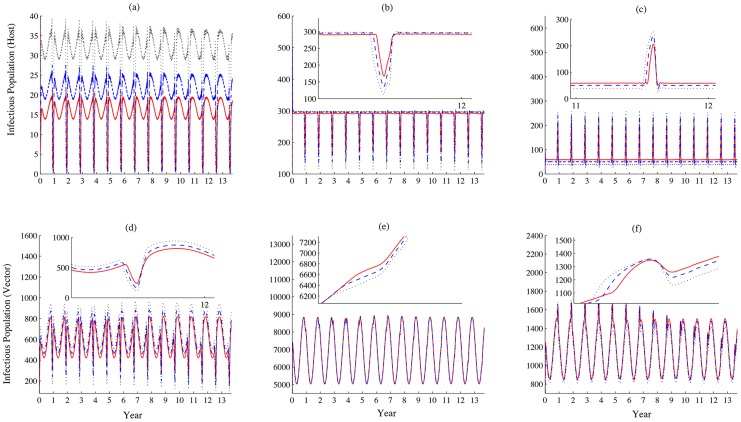
How the starting time of festival preparation impacts patterns of disease outbreaks: infectious classes. (a)-(c) and (d)-(f), simulations of the populations of infectious livestock and vectors in patches 1–3, respectively. The starting time of festival preparation varies from 2, 3, to 4 weeks ago (n = 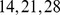 days), represented by solid, dashed and dotted lines, respectively. Values of other parameters are identical with those used in Fig. 3 and 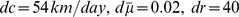. Unit: daily. When the preparation starts early, we are expecting a larger scale of disease outbreaks due to the higher concentration of livestock, larger scale infectious population appear in patches 1 and 3. However, less number of infectious individuals exist due to the exposed period in patch 2.

**Figure 12:**
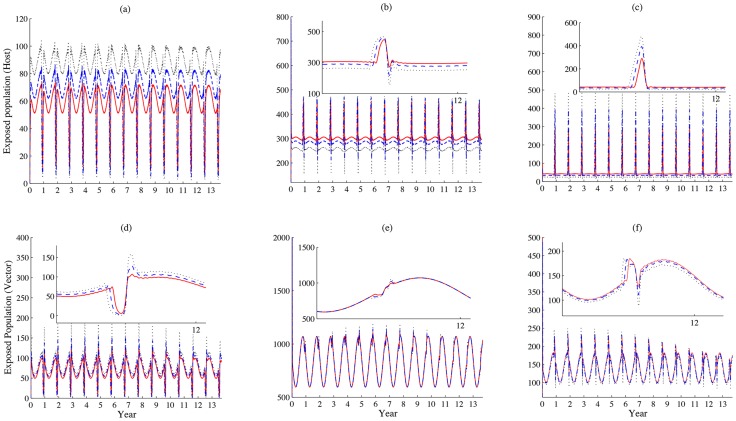
How the starting time of festival preparation impacts patterns of disease outbreaks: exposed classes. (a)-(c) and (d)-(f), simulations of the populations of exposed livestock and vectors in patches 1–3, respectively. The starting time of festival preparation varies from 2, 4, to 6 weeks ago (n = 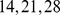 days), represented by solid, dashed and dotted lines, respectively. Values of other parameters are identical with those used in Fig. 3 and 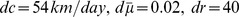. Unit: daily. When the preparation starts early, we are expecting a larger scale of disease outbreaks due to the higher concentration of livestock. Therefore, more individuals in exposed period we can observe when the starting time varies from 2 to 6 weeks ago.

Drake et al. [1] pointed out that the festival activities may even begin two months ahead of Eid al-Adha. We also vary the starting time for festival preparation to estimate its impacts on disease dynamics. It is observed that when the activities start earlier, the size of infectious livestock population decreases in patch 1, but the population increases in patch 3. The early preparation for the festival leads to high concentration of livestock at the location where the festival will be held, and results in huge disease outbreaks locally. We find that more individuals becomes infected in each patch during the festival (see Fig. 11 (a), (c) and Fig. 12 (a)-(c)), but more individuals experience their exposed period than infectious period when they travel in patch 2 (see Fig. 11 (b)).

## Discussion

We investigated the festival-driven and seasonal impacts on the patterns of RVF outbreaks among livestock in Africa and Middle East. Although we did not consider the compartments for humans directly in our model, human activities were reflecting by those periodic parameters, such as the importation and transportation (i.e., movement speed) of livestock in these regions, and have various impacts on the patterns of disease outbreaks at different locations along the transportation route. From the analysis and simulations of the model, we found that importation of livestock results in more livestock and increases the local reproduction number in patch 1, the transportation of animals from patch 1 to the other patches reduces the chance of disease outbreaks. Also, the disease spreads to other patches due to the movement of livestock. Not surprisingly, when the time for festival preparation starts earlier (an expectation of Islam on a large scale), the risk of massive disease outbreaks rises, particularly in patch 3 (the Nile Delta).

In order to understand the critical parameters in the spread of the disease and transmission of the disease pathogen, we also varied the daily increments in the number of imported animals and the movement speed of livestock during the festival. From Fig. 10 (a)-(c), we observed that the yearly cumulative number of infected livestock in patch 1 was influenced by both critical parameters, while the impact from daily increment in the daily imported number dominates in the other two patches, particularly patch 2. Since patch 2 is a transitional patch, in which livestock move in and out, the impact of movement is not significant. A model with more than three patches, i.e. a four-patch model, was examined as well, and we do not find a qualitatively different pattern from that of the three-patch model by adding more transitional patches. Therefore, we did not present the numerical results for the four-patch model. In addition, the periodic movement has significantly changed the daily numbers of the infected population, but will not impact the cumulative infected population size too much. Even if those infected livestock will move in or out quickly, they are still counted in the yearly cumulative infected numbers in each patch.

The disease persists in at least one patch, as the global basic reproduction number is greater than one, while the coincidence of the high densities of livestock and vectors (with the greatest overlap occurring every 33 years) will increase the likelihood of a RVF outbreak (Fig. 6 (a)-(f)). The dependencies of local or global reproduction numbers on the increments in the daily imported number of livestock and the timing of festival preparation exhibit similar patterns as those shown in Fig. 8 (a)-(d).

In this work, the interaction between effects of seasonality and the social economy is mathematically confirmed to be a major factor of the local disease outbreaks and transmissions of the disease pathogen, which is different from the disease transmission mechanisms in other regions of Africa. For years, the vertical transmission of the disease pathogen through infected Aedes mosquito eggs have been believed to be the major cause of the long-term persistence of the disease in West Africa [10], [21], [49]. Investigators performed risk assessment statistically or mathematically, trying to identify reasons for disease outbreaks in Egypt, as the Aedes mosquito is not a commonly reported species locally. Researchers have considered seasonality and socioeconomic impacts individually [1], [17]. The patch model we proposed in the paper incorporates both effects related to different calendars and reveals the sporadic epidemic/epizootic that occurred in Egypt since 1977. Abundances of hosts and vectors increase the probability of large disease outbreaks. For example, an unexpected RVF outbreak during September to October, 2010, was attributed to a large number of camels, which played the role of hosts in northern Mauritania, along with exceptionally heavy rainfall [50]. With these findings, we are in principle able to provide some information to local governments on how to correctly predict the disease outbreaks and how to effectively control the transmission of the disease pathogen. Reducing the abundance of vectors is a possible approach. Preventive measures may be taken during the importation and transportation of livestock. Actions considering both impacts from seasonality and social economy will achieve a better result in disease control.

Though our simulations (Fig. 6 (a)-(f)) show that the timeline of disease outbreaks on a larger scale still follows a certain pattern, which does not completely match the real scenario; i.e., sporadic outbreaks of RVF in Egypt. This may be explained by the following factors. Since we assumed well-mixed populations of livestock in each patch and identical movement rates regardless the clinical stage of livestock during transportation, the model may overestimate the real situation. In practice, infected livestock may be removed once they are identified, and therefore are not be a source of infection any longer. Implementations of various kinds of vaccination strategies and interventions of other stochastic events, such as unpredicted religious festival preparations, will also change the pattern of disease outbreaks. Further, we may extend our model to consider the disease latencies in both hosts and vectors, and the importation of livestock carrying RVFV during disease latency, as these factors also impact the pattern of disease outbreaks.

The force of infection in our paper is in term of pseudo-mass action transmission [41], as we assumed that the effective biting rate is linearly-density dependent 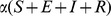. But for the vector-borne disease models, it is more common to adopt the true-mass action transmission mechanism, where the effective biting rate is a constant and the force of infection is proportional to the density of the infectious host population when the disease pathogen transmitted from livestock to mosquito (the susceptible host population when the disease pathogen transmitted from mosquito to livestock). The mathematical analysis on our model will not be affected by taking the new force of infection, but the results of numerical simulations may change. We would extent our model considering this type of force of infection in our future work.

## Supporting Information

S1 Text (PDF)
