# Supplementary material for: Modelling the Effects of Seasonality and Socioeconomic Impact on the Transmission of Rift Valley Fever Virus
Source: PLoS Negl Trop Dis. 2015 Jan 8;9(1):e3388. doi: 10.1371/journal.pntd.0003388 (PMC4287488; doi:10.1371/journal.pntd.0003388)
Supplement: S1 Text — (PDF) [file pntd.0003388.s001.pdf]

Supporting Text

Modelling the Effects of Seasonality and Socioeconomic Impact on the Transmission of Rift Valley Fever Virus

Yanyu Xiao[1,2], John C. Beier[2], Robert Stephen Cantrell[1], Chris Cosner[1], Donald L. DeAngelis[3], Shigui Ruan[1,*]

1 Department of Mathematics, University of Miami, Coral Gables, FL 33124, USA

2 Department of Public Health Science, Miller School of Medicine, University of Miami, Miami, FL 33136, USA

3 U.S. Geological Survey, Department of Biology, University of Miami, Coral Gables, FL 33124, USA

*E-mail: ruan@math.miami.edu

Appendix: Mathematical analysis

We put the mathematical analysis of the system (2) in this appendix. The well-posedness of the model

$$
\left\{
\begin{aligned}
\frac{\mathrm{d}S_1}{\mathrm{d}t} =& r(t) - \alpha_1 S_1 V_1 - \mu S_1 + \zeta R_1 - \frac{C(t)}{d_1} S_1,\\
\frac{\mathrm{d}E_1}{\mathrm{d}t} =& \alpha_1 S_1 V_1 - (\mu + \epsilon_1) E_1 - \frac{C(t)}{d_1} E_1,\\
\frac{\mathrm{d}I_1}{\mathrm{d}t} =& \epsilon_1 E_1 - (\mu + \gamma + \delta) I_1 - \frac{C(t)}{d_1} I_1,\\
\frac{\mathrm{d}R_1}{\mathrm{d}t} =& \gamma I_1 - (\mu + \zeta) R_1 - \frac{C(t)}{d_1} R_1,\\
\frac{\mathrm{d}U_1}{\mathrm{d}t} =& \xi_1 (U_1 + L_1 + V_1) - \frac{\xi_1 - \nu_1}{M_1(t)} (U_1 + L_1 + V_1)^2 - \nu_1 U_1 - \beta_1 I_1 U_1,\\
\frac{\mathrm{d}L_1}{\mathrm{d}t} =& -(\nu_1 + \eta_1) L_1 + \beta_1 I_1 U_1,\\
\frac{\mathrm{d}V_1}{\mathrm{d}t} =& -\nu_1 V_1 + \eta_1 L_1,
\end{aligned}
\right.
$$

$$
\left\{
\begin{aligned}
\frac{\mathrm{d}S_2}{\mathrm{d}t} =& \frac{C(t)}{d_1} S_1 - \alpha_2 S_2 V_2 - \mu S_2 + \zeta R_2 - \frac{C(t)}{d_2} S_2,\\
\frac{\mathrm{d}E_2}{\mathrm{d}t} =& \frac{C(t)}{d_1} E_1 + \alpha_2 S_2 V_2 - (\mu + \epsilon_2) E_2 - \frac{C(t)}{d_2} E_2,\\
\frac{\mathrm{d}I_2}{\mathrm{d}t} =& \frac{C(t)}{d_1} I_1 + \epsilon_2 E_2 - (\mu + \gamma + \delta) I_2 - \frac{C(t)}{d_2} I_2,\\
\frac{\mathrm{d}R_2}{\mathrm{d}t} =& \frac{C(t)}{d_1} R_1 + \gamma I_2 - (\mu + \zeta) R_2 - \frac{C(t)}{d_2} R_2,\\
\frac{\mathrm{d}U_2}{\mathrm{d}t} =& \xi_2 (U_2 + L_2 + V_2) - \frac{\xi_2 - \nu_2}{M_2(t)} (U_2 + L_2 + V_2)^2 - \nu_2 U_2 - \beta_2 I_2 U_2,\\
\frac{\mathrm{d}L_2}{\mathrm{d}t} =& -(\nu_2 + \eta_2) L_2 + \beta_2 I_2 U_2,\\
\frac{\mathrm{d}V_2}{\mathrm{d}t} =& -\nu_2 V_2 + \eta_2 L_2,
\end{aligned}
\right. \tag{2}
$$

$$
\left\{
\begin{aligned}
\frac{\mathrm{d}S_3}{\mathrm{d}t} =& \frac{C(t)}{d_2} S_2 - \alpha_3 S_3 V_3 - \bar{\mu}(t) S_3 + \zeta R_3,\\
\frac{\mathrm{d}E_3}{\mathrm{d}t} =& \frac{C(t)}{d_2} E_2 + \alpha_3 S_3 V_3 - (\bar{\mu} + \epsilon_3) E_3,\\
\frac{\mathrm{d}I_3}{\mathrm{d}t} =& \frac{C(t)}{d_2} I_2 + \epsilon_3 E_3 - (\bar{\mu}(t) + \gamma + \delta) I_3,\\
\frac{\mathrm{d}R_3}{\mathrm{d}t} =& \frac{C(t)}{d_2} R_2 + \gamma I_3 - (\bar{\mu}(t) + \zeta) R_3,\\
\frac{\mathrm{d}U_3}{\mathrm{d}t} =& \xi_3 (U_3 + L_3 + V_3) - \frac{\xi_3 - \nu_3}{M_3(t)} (U_3 + L_3 + V_3)^2 - \nu_3 U_3 - \beta_3 I_3 U_3,\\
\frac{\mathrm{d}L_3}{\mathrm{d}t} =& -(\nu_3 + \eta_3) L_3 + \beta_3 I_3 U_3,\\
\frac{\mathrm{d}V_3}{\mathrm{d}t} =& -\nu_3 V_3 + \eta_3 L_3.
\end{aligned}
\right.
$$

is guaranteed by the following Theorem,

Theorem 1 *For any nonnegative initial condition* $\mathbf{X} = (\mathbf{S(0)}, \mathbf{E(0)}, \mathbf{I(0)}, \mathbf{R(0)}, \mathbf{U(0)}, \mathbf{L(0)}, \mathbf{V(0)}) \in \mathbb{R}_+^{\mathbf{21}}$, *where* $S(0) = (S_1(0), S_2(0), S_3(0))$, *with analogous forms* E, I, R, U, L, V, *system* (2) *admits a unique solution in* $\mathbb{R}_+^{21}$ *and is bounded for* $t \geq 0$.

The proof of the theorem follows by showing the boundedness of the population sizes of livestock and female mosquitoes, respectively.

In absence of the disease, system (2) reduces to

$$\begin{aligned} S_1'(t) =& r(t) - (\mu + \frac{C(t)}{d_1})S_1(t) := F_1(t, S_1, S_2, S_3), \\ S_2'(t) =& \frac{C(t)}{d_1}S_1(t) - (\mu + \frac{C(t)}{d_2})S_2(t) := F_2(t, S_1, S_2, S_3), \\ S_3'(t) =& \frac{C(t)}{d_2}S_2(t) - \bar{\mu}(t)S_3(t) := F_3(t, S_1, S_2, S_3), \end{aligned} \tag{A1}$$

and

$$\begin{aligned} U_1'(t) =& \xi_1 U_1 - \frac{\xi_1 - \nu_1}{M_1(t)}U_1^2 - \nu_1 U_1, \\ U_2'(t) =& \xi_2 U_2 - \frac{\xi_2 - \nu_2}{M_2(t)}U_2^2 - \nu_2 U_2, \\ U_3'(t) =& \xi_3 U_3 - \frac{\xi_3 - \nu_3}{M_3(t)}U_3^2 - \nu_3 U_3. \end{aligned} \tag{A2}$$

It is easy to see that system (A1) can be solved equation by equation and that the equations in system (A2) are decoupled. Moreover, the solutions of system (A1) are positively invariant in $\mathbb{R}_+^3$. Denote $F(t, S) := (F_1(t, S_1, S_2, S_3), F_2(t, S_1, S_2, S_3), F_3(t, S_1, S_2, S_3))$. $F(t, S)$ is a subhomogeneous mapping for $S \in \mathbb{R}_+^3$, in the sense that $F(t, \alpha S) >> \alpha F(t, S)$, $\alpha \in (0, 1)$ for any $t \geq 0$. Analogous to Theorem 1, we can obtain the ultimate boundedness of the solution of (A1). Solving the system (A1) equation by equation, we obtain

$$S_1(t) = e^{-\int_0^t (\mu + \frac{C(s)}{d_1})\,\mathrm{d}s} \left(S_1(0) + \int_0^t r(s) e^{\int_0^s (\mu + \frac{C(\xi)}{d_1})\,\mathrm{d}\xi}\,\mathrm{d}s \right).$$

We get a periodic solution $S_1^*(0)$ of period T_1 via the requirement that $S_1^*(T_1) = S_1^*(0)$, leading to

$$S_1^*(0) = \frac{\int_0^{T_1} r(s) e^{\int_0^s (\mu + \frac{C(\xi)}{d_1})\,\mathrm{d}\xi}\,\mathrm{d}s}{e^{\int_0^{T_1} (\mu + \frac{C(s)}{d_1})\,\mathrm{d}s} - 1}.$$

We then may obtain the expression of $S_2^*(t)$ by substituting the solution of $S_1^*(t)$ into the second equation, and similarly for $S_3^*(t)$. System (A1) admits a unique positive periodic solution (period is T_1), which is globally asymptotically stable in $\mathbb{R}_+^3$ [1, 2]. Solving equations in system (A2) individually, we can also get the global stability of the positive periodic solution $U_1^*(t)$, $U_2^*(t)$, $U_3^*(t)$ (period is T_2). Denote the disease free state as $E^0 = (S_1^*(t), S_2^*(t), S_3^*(t), U_1^*(t), U_2^*(t), U_3^*(t))$.

Since we have two periods T_1 and T_2 involved in our model, we have an almost periodic system. Following [3–5], we linearize all the equations for infectious compartments $z = (E_1,\ I_1,\ E_2,\ I_2,\ E_3,\ I_3,\ L_1,\ V_1,\ L_2,\ V_2,\ L_3,\ V_3)^T$ of system (2) at the disease free state. The related Jacobian matrix can be written as

$$J|_{E^0} = F(t) - V(t),$$

where

$$F(t) = \begin{pmatrix}
0 & 0 & 0 & 0 & 0 & 0 & 0 & \alpha_1 S_1^*(t) & 0 & 0 & 0 & 0 \\
0 & 0 & 0 & 0 & 0 & 0 & 0 & 0 & 0 & 0 & 0 & 0 \\
0 & 0 & 0 & 0 & 0 & 0 & 0 & 0 & 0 & \alpha_2 S_2^*(t) & 0 & 0 \\
0 & 0 & 0 & 0 & 0 & 0 & 0 & 0 & 0 & 0 & 0 & 0 \\
0 & 0 & 0 & 0 & 0 & 0 & 0 & 0 & 0 & 0 & \alpha_3 S_3^*(t) & \\
0 & 0 & 0 & 0 & 0 & 0 & 0 & 0 & 0 & 0 & 0 & 0 \\
0 & \beta_1 U_1^*(t) & 0 & 0 & 0 & 0 & 0 & 0 & 0 & 0 & 0 & 0 \\
0 & 0 & 0 & 0 & 0 & 0 & 0 & 0 & 0 & 0 & 0 & 0 \\
0 & 0 & 0 & \beta_2 U_2^*(t) & 0 & 0 & 0 & 0 & 0 & 0 & 0 & 0 \\
0 & 0 & 0 & 0 & 0 & 0 & 0 & 0 & 0 & 0 & 0 & 0 \\
0 & 0 & 0 & 0 & 0 & \beta_3 U_3^*(t) & 0 & 0 & 0 & 0 & 0 & 0 \\
0 & 0 & 0 & 0 & 0 & 0 & 0 & 0 & 0 & 0 & 0 & 0
\end{pmatrix},$$

and

$$V(t) = \begin{pmatrix} V_1 & \mathbf{0} \\ \mathbf{0} & V_2 \end{pmatrix},$$

and

$$V_1 = \begin{pmatrix} \mu+\epsilon_1+\frac{C(t)}{d_1} & 0 & 0 & 0 & 0 & 0 \\ -\epsilon_1 & \mu+\gamma+\delta+\frac{C(t)}{d_1} & 0 & 0 & 0 & 0 \\ -\frac{C(t)}{d_1} & 0 & \mu+\epsilon_2+\frac{C(t)}{d_2} & 0 & 0 & 0 \\ 0 & -\frac{C(t)}{d_1} & -\epsilon_2 & \mu+\gamma+\delta+\frac{C(t)}{d_1} & 0 & 0 \\ 0 & 0 & -\frac{C(t)}{d_2} & \mu+\epsilon_3+\frac{C(t)}{d_3} & 0 & 0 \\ 0 & 0 & 0 & -\frac{C(t)}{d_2} & -\epsilon_3 & \mu+\gamma+\delta+\frac{C(t)}{d_3} \end{pmatrix},$$

and

$$V_2 = \begin{pmatrix} \eta_1+\nu_1 & 0 & 0 & 0 & 0 & 0 \\ -\eta_1 & \nu_1 & 0 & 0 & 0 & 0 \\ 0 & 0 & \eta_2+\nu_2 & 0 & 0 & 0 \\ 0 & 0 & -\eta_2 & \nu_2 & 0 & 0 \\ 0 & 0 & 0 & 0 & \eta_3+\nu_3 & 0 \\ 0 & 0 & 0 & 0 & -\eta_3 & \nu_3 \end{pmatrix}.$$

Let $Y(t,s), t \geq 0$ be the evolution operator of the almost periodic system

$$\frac{\mathrm{d}y(t)}{\mathrm{d}t} = V(t)y(t).$$

Then, for each $s \in \mathbb{R}$, the 12×12 matrix $Y(t,s)$ satisfies

$$\frac{\mathrm{d}Y(t,s)}{\mathrm{d}t} = -V(t)Y(t,s), t \geq s, Y(s,s) = I_{6\times 6},$$

where $I_{12\times 12}$ is the 12×12 identity matrix.

Define

$$AP(F,V) := \{\phi : \phi \in AP(\mathbb{R}, \mathbb{R}^{12}), mod\,\phi \subset mod(F,\, V)\},$$

By Lemma 2.1 in [4], $AP(F,V)$ is a Banach space of all almost periodic functions from $\mathbb{R}$ to $\mathbb{R}^{12}$, with the maximum norm. $mod\,f(t)$ is defined as the smallest additive group of real numbers that contains the Fourier exponent of $f(t)$, where $f(t) \in AP(F,V)$ is an almost periodic function [6]. Assume $\phi(s)$ is the initial distribution of all the infectious compartments. Define a linear operator $L : AP(F,V) \to AP(F,V)$

by

$$\begin{aligned}(L\phi)(t) &= \int_{-\infty}^{t} Y(t,s)F(s)\phi(s)\,\mathrm{d}s \\ &= \int_{0}^{\infty} Y(t,t-a)F(t-a)\phi(s-a)\,\mathrm{d}a, \quad t \in \mathbb{R}, \phi \in AP(F,V).\end{aligned}$$

It is easy to verify the conditions (A1)-(A7) in Wang and Zhao [4]. Hence, the basic reproduction number of the almost periodic system (2) can be defined as $R_0 = \rho(L)$, and L is the so-called next generation operator. In [3], a next generation operator for periodic systems is derived, we can get the same formula for R_0 if we consider our system as a periodic system with a period $T = lcm(T_1, T_2)$. In [7], another linear operator defined as $\bar{L} = \int_0^\infty F(t)Y(t,t-1)\phi(t,a)\,\mathrm{d}a$ has spectral radius which is equal to $\rho(L)$. The complexity of the model prevents us from obtaining the explicit form of basic reproduction number. Although R_0 provides the information on whether the disease will eventually die out in the three patches or not, we actually pay more attention to the *instantaneous basic reproduction number*, computed by the recipe of the next generation matrix [8] based on the number of susceptible population and values of parameters at the current time. The instantaneous basic reproduction number for three patches or an individual patch gives detailed and immediate changes of infections at different times during one year or period.

References

1. Zhang T, Teng Z (2007) On a nonautonomous SEIRS model in epidemiology. Bull Math Biol 69: 2537-2559.

2. Gao D, Lou Y, Ruan S (2014) A periodic Ross-Macdonald model in a patchy environment. Discrete Contin Dyn Syst Ser B, in press .

3. Wang W, Zhao XQ (2008) Threshold dynamics for compartmental epidemic models in periodic environments. J Dyn Differ Equ 20: 699-717.

4. Wang B, Zhao XQ (2013) Basic reproduction ratios for almost periodic compartmental epidemic models. J Dyn Differ Equ 25: 535-562.

5. Diekmann O, Heesterbeek JAP, Roberts MG (2010) The construction of next-generation matrices for compartmental epidemic models. J Royal Soc Interface 7: 873-885.

6. Fink A (1974) Almost Periodic Differential Equations. Lecture Notes in Mathematics. Springer, Berlin.

7. Bacaër N (2007) Approximation of the basic reproduction number r_0 for vector-borne diseases with a periodic vector population. Bull Math Biol 69: 1067-1091.

8. van den Driessche P, Watmough J (2002) Reproduction numbers and sub-threshold endemic equilibria for compartmental models of disease transmission. Math Biosci 285: 29-48.
